# Lipid Droplets: A Cellular Organelle Vital for Thermogenesis

**DOI:** 10.7150/ijbs.77051

**Published:** 2022-10-24

**Authors:** Lupeng Chen, Yi Jin, Jian Wu, Zhuqing Ren

**Affiliations:** 1 Key Laboratory of Agriculture Animal Genetics, Breeding and Reproduction of the Ministry of Education, College of Animal Science and Technology, Huazhong Agricultural University, Wuhan 430070, China.; 2 Hubei Hongshan Laboratory, Wuhan, China.

**Keywords:** Lipid droplets, Thermogenesis, Brown/beige adipose tissue, Lipophagy

## Abstract

Mammals maintain a constant core body temperature through adaptive thermogenesis which includes shivering and non-shivering thermogenesis. Non-shivering thermogenesis relies primarily on mitochondrial uncoupling protein 1 (UCP1) in thermogenic fat (including brown and beige adipose tissue) to burn substrates, such as fatty acids (FAs), and convert chemical energy into heat. Lipid droplets (LDs), which are organelles that store lipids, are present in large numbers in thermogenic fat and are essential for adipose thermogenesis. Upon cold stimulation, LDs rapidly release FAs through autophagy or lipase-mediated lipolysis and rapidly translocate FAs into the mitochondria by interacting with mitochondria to burn and so promote thermogenesis. In addition, LD proteins promote the expression of UCP1 by activating the transcriptional activity of thermogenesis-related proteins. Here, the progress of research on the important role of LDs in thermogenesis is reviewed, mainly in terms of LD proteins, LD-organelle interactions, and LD autophagy (lipophagy). The emerging rationale for the involvement of LDs in each thermogenic pathway is described and the remaining unanswered questions in this field are highlighted.

## Introduction

Adiposes are often classified into thermogenic and non-thermogenic fats, which define their thermogenic capability 1, 2. Non-thermogenic fat is represented by white adipose tissue 3 (WAT), which is characterized by large unilocular lipid droplets (LDs) and a low range of mitochondria with low levels of uncoupling protein 1 (UCP1). White adipose tissue is responsible for the body's energy storage by storing it in unilocular LD as triglycerides. In contrast, thermogenic adipose includes brown adipocytes and beige adipocytes 4, which are characterized by multilocular LDs and mitochondria with high-density cristae structures expressing UCP1 that can efficiently consume energy in the form of heat 5.

Mammals maintain a constant core body temperature through adaptive thermogenesis which includes shivering and non-shivering thermogenesis 6, 7 in response to cold stimuli. Thermogenic adipose tissue plays a key role in non-shivering thermogenesis in the form of an ineffective enzymatic cycle 8 or by relying on UCP1 to deregulate respiration during ATP synthesis and consuming chemical energy to form heat. The sources of energy for non-shivering thermogenesis are diverse and include glucose 6, 9, 10, fatty acids (FAs) 11, succinate 12 and branched-chain amino acids 13, 14. Although there are multiple options for energy sources in thermogenesis, FAs are the primary molecules selected for adipose thermogenesis. In response to cold stimulation, the adipose tissue thermogenesis program is activated, and LDs in adipocytes release FAs by lipases, which is burned as fuel by the mitochondria to produce heat. This may explain why brown and beige adipocytes contain large amounts of LDs. To determine how adipocytes regulate lipid metabolism, such as FA metabolism, to generate heat, it is necessary to understand the important organelles that store, transfer, and metabolize lipids in LDs.

## Generation and growth of lipid droplets in thermogenic fat

**Figure 1:**
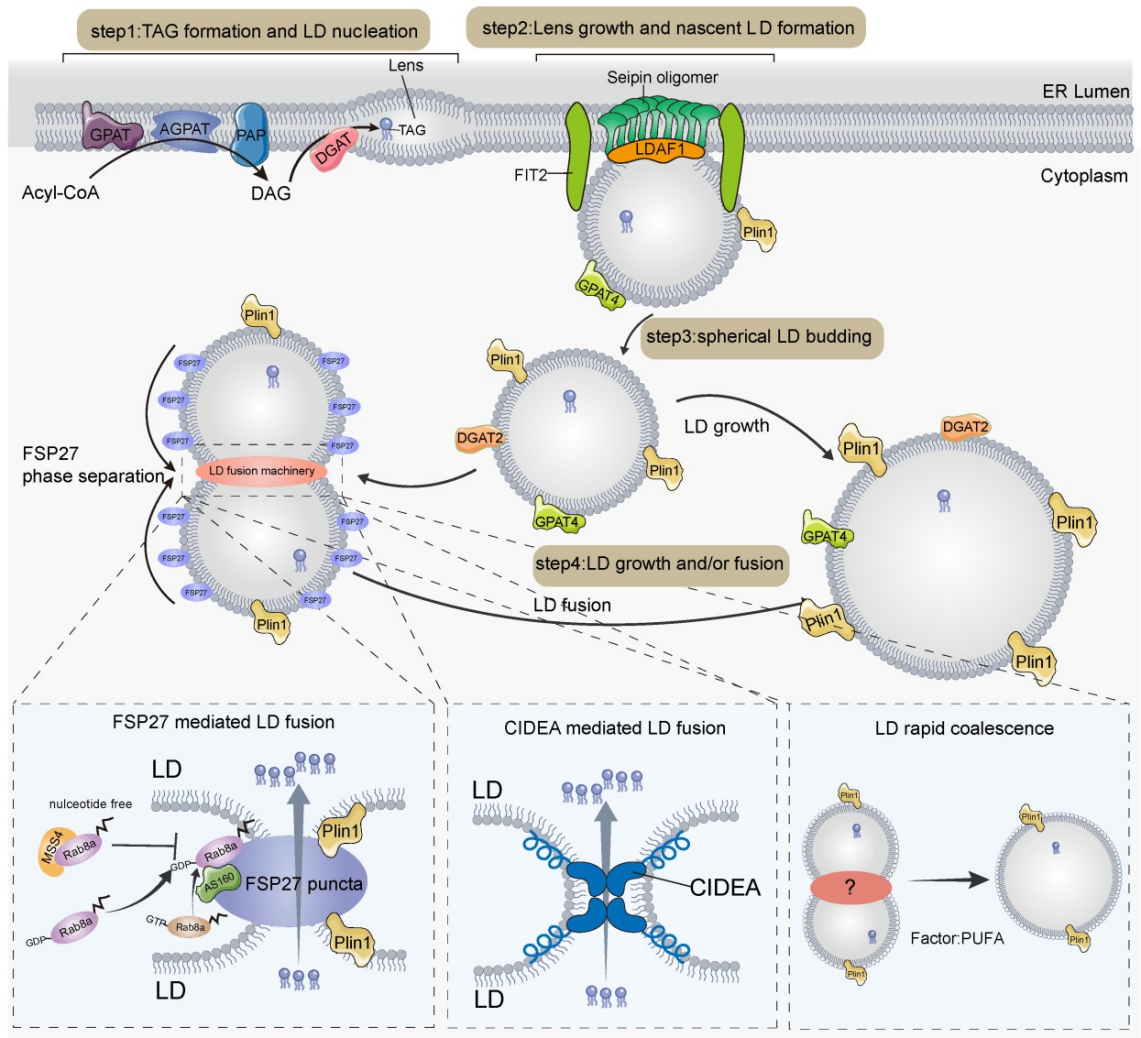
** Model of lipid droplet generation, growth, and fusion in adipose tissue.** ER, endoplasmic reticulum; LLPS, liquid-liquid phase separation; TAG, Triglyceride; Plin1, perilipin1; AGPAT, acylglycerolphosphate acyltransferase; DAG, diacylglycerol; DGAT2, Acyl-CoA: diacylglycerol acyltransferase 2; GPAT4, glycerol-3 phosphate acyltransferase 4; CIDE, cell death-inducing DFF45-like effector; PAP, phosphatidic acid phosphatase; LDCS, LD contact sites; GTP, guanosine triphosphate; GDP, guanosine diphosphate; PUFA, polyunsaturated fatty acids.

LDs are widespread dynamic organelles that consist of a monolayer of phospholipids encapsulated in neutral lipids, with a variety of specific LD-associated proteins 15-17. LDs are found in a variety of tissues in animals, including the heart, liver, kidney, and adipose tissue, with thermogenic fat being the tissue containing a large number of LDs, demonstrating the importance of LDs in adipose tissue. The biogenesis of LDs occurs in the endoplasmic reticulum (ER), in a highly regulated biological process 15, 16, 18. First, FAs and coenzyme A (CoA) are catalyzed by acyl CoA ligases to form acetyl CoA, whose acyl groups are transferred to diacylglycerol (DAG), cholesterol, and eventually triglyceride (TAG) and cholesteryl ester (CE) catalyzed by Acyl-CoA: diacylglycerol acyltransferases (DGATs) and acyl-CoA: cholesterol acyltransferase enzymes (ACATs) in the ER membrane 17; following this, the neutral lipids undergo liquid-liquid phase separation and nucleation 19. Second, the nucleated neutral lipid domains grow and bud towards the cytoplasmic side by the action of seipin 20-24, lipid droplet assembly factor 1 (LDAF1) 25, 26, fat storage-inducing transmembrane protein 2 (FIT2) 27, 28, and other factors, and are finally shed from the ER. Third, a variety of lipid synthases comprising the newly generated LDs, such as GPAT4 29-31 and DGAT2 32, catalyze the formation of TAG directly on the surface of LDs and thus promote the expansion of LDs (**Figure 1**).

In addition, LDs can fuse to form larger droplets in response to cell death-inducing DFF45-like effector (CIDE) family proteins 33. CIDE family proteins consist of CIDEA, CIDEB, and CIDEC (also known as fat-specific protein 27 (FSP27)) are highly expressed in adipose tissue, suggesting that LD fusion is essential for maintaining the morphology and stability of LDs in adipose tissue 34. CIDE is homogeneously distributed on the LD surface, but upon fusion of LDs, liquid-liquid phase separation occurs and rapidly accumulates at LD contact sites (LDCS). This forms highly plastic and lipid-permeable fusion plates that contain unique lipid channels that allow neutral lipid flow from donor to recipient LDs, thereby mediating atypical LD fusion 35, 36. CIDE proteins regulate LD fusion by recruiting other regulators to LD contact sites; for example, CIDEC recruits Rab8a and Plin1 to positively regulate LD fusion activity 37, 38 (**Figure 1**). Among them, plin1 can promote CIDEC-mediated LD fusion by expanding pore size or increasing LD surface tension 37. Rab8a-GDP but not Rab8a-GTP binds to the C-terminal structural domain of CIDEC, which leads to the recruitment of Rab8a to the LDCS and the formation of a complex with Rab8a's GTPase-activating protein (GAP) AS160 and CIDEC 38, thereby promoting LD fusion and growth. Another family member that regulates LD fusion is CIDEA 33, 39-41. CIDEA is specifically highly expressed in brown adipose tissue and promotes LD fusion during adipose differentiation 41. The amphipathic helix in CIDEA is essential for LD fusion, which helps CIDEA to embed in the LD phospholipid monolayer and bind phosphatidic acid (PA). The N-terminal structural domain of CIDEA docks with the C-terminal dimerization region of the trans-complex and is enriched in the LDCS, interacting with the cone-shaped phospholipid PA, which may increase the permeability of the phospholipid barrier and facilitate LD fusion through lipid transfer 39. In the liver, CIDEB localizes to LDCS and promotes lipid exchange and LD fusion in small LD-containing hepatocytes and large LD-containing hepatocytes 42. In contrast, it is unknown whether CIDEB regulates LD fusion in adipocytes, as CIDEB is mainly expressed in the liver and kidney 43.

In white adipocytes, LDs eventually fuse to form large unilocular LDs, which occupy most of the cellular space 35, 36. However, in brown or beige adipocytes, LDs are numerous and much smaller in diameter than the large unilocular LDs of white adipocytes, and the small multilocular LDs are essential for the efficient flow of hydrolyzed free fatty acids (FFA) into neighboring mitochondria for β-oxidation 44 and heat production. This implies that the LDs of thermogenic adipocytes cannot fuse indefinitely at room temperature or cold conditions; therefore, a mechanism is required to inhibit the indefinite fusion of LDs. MSS4, an atypical guanine nucleotide exchange factor (GEF) of Rab8a, maintains Rab8a in a nucleotide-free state by converting Rab8a-GDP to Rab8a-GTP, which allows separation of LD contact sites and thus inhibits CIDEC-mediated LD fusion 38. In brown adipose tissue, there is an FSP27 isoform, FSP27β, whose deletion leads to a larger diameter and a smaller number of LDs with a white adipocyte phenotype 45. Mechanistically, FSP27β can form a complex with CIDEA in brown adipose tissue and inhibit the formation of CIDEA dimers, thus inhibiting CIDEA from regulating the fusion of LDs in brown adipocytes, ensuring the stable presence of smaller LDs in brown adipocytes, and avoiding excessive fusion of LDs 45, 46. In addition, in white fat, histone deacetylase 6 (HDAC6) deacetylates CIDEC, leading to CIDEC destabilization, thereby reducing LD fusion. In contrast, FAs, by promoting the dissociation of CIDEC from HDAC6, can prevent CIDEC deacetylation and thus promote LD fusion 47.

LDs are organelles generated from the ER. The generation of LDs is divided into processes including nucleation, nascent LD formation, budding and maturation. First, FAs are catalyzed by a series of enzymes to catalyze the synthesis of TAG, which forms lens structures in the ER by LLPS. Second, lens grows and forms nascent LD driven by seipin, LDAF1, FIT2, and other factors. Third, spherical LD that have grown to a particular stage buds out from the ER. Finally, further maturation of LDs by growth or fusion. DGAT2 and GPAT4 on the surface of LDs promote the growth and expansion of LDs through local lipid synthesis. LD fusion is mediated by CIDE proteins. CIDEC aggregates with LDCS by LLPS and regulates LD fusion via interactions with Plin1, AS160, and Rab8a. CIDEA promotes LD fusion by forming a trans-complex in LDCS. In addition, LDs can also become larger by transient fusion and PUFA facilitates this process, in which it has not been determined whether proteins are involved.

## The role of lipid droplet proteins in adipose thermogenesis

**Figure 2:**
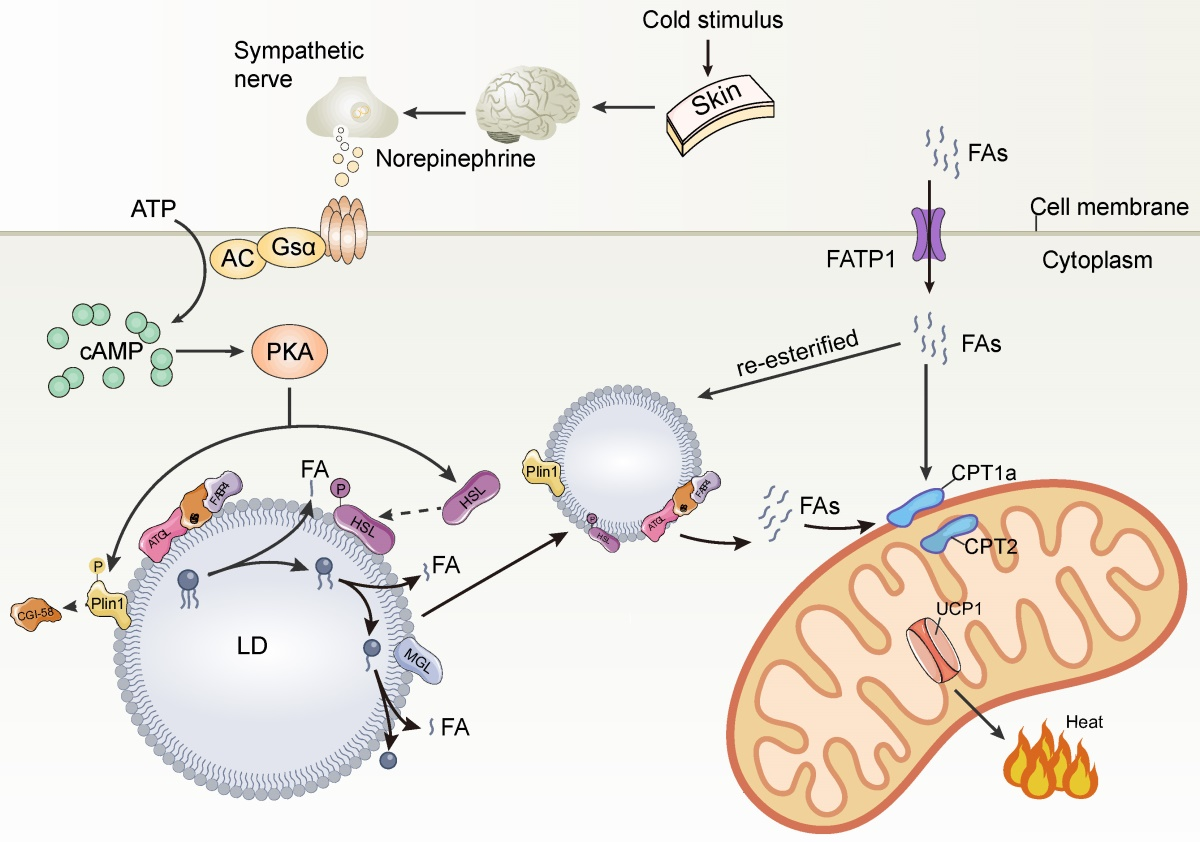
**Cold stimulation-mediated lipid droplet breakdown and thermogenesis.** AC, adenylate cyclase; PKA, protein kinase A; CGI-58, comparative gene identification-58; Plin1, perilipin1; ATGL, adipose triglyceride lipase; FABP4: fatty acid binding protein 4; HSL, hormone-sensitive lipase; cAMP, cyclic adenosine monophosphate; MGL, monoacylglycerol lipase; FATP1: fatty acid transport protein 1; CPT1a, carnitine palmitoyltransferase 1A; CPT2, carnitine palmitoyltransferase 2.

LDs are coated with various proteins, including members of the PLIN family and lipolytic enzymes, which are essential for LD function. The most important role of LDs in thermogenic lipids is to maintain body temperature by rapidly producing FAs for mitochondrial burning and heat generation in response to cold stimulation. The rapid mobilization of lipids depends on the lipolytic enzymes adipose triglyceride lipase (ATGL) and hormone-sensitive lipase (HSL) on the LD surface. Under cold stimulation conditions, the skin transmits the perceived cold signal to the hypothalamus, which activates brown adipose tissue (BAT) via norepinephrine (NA) released from the sympathetic nerve endings. Norepinephrine (NA) binds to β-adrenoceptors (β-ARs) and then to Gα G-proteins that activate adenylate cyclase (AC), thereby increasing cellular cyclic adenosine monophosphate (cAMP) concentrations. cAMP further activates protein kinase A (PKA), leading to phosphorylation of Plin1, which in turn activates ATGL 48, 49. ATGL hydrolyzes triglycerides stored in LDs to DAG and FFA, which is the rate-limiting step in triglyceride hydrolysis. HSL phosphorylated by PKA then hydrolyzes DAG to FFA and monoacylglycerol, which can be hydrolyzed to glycerol and FFA by HSL, MGL, and ABHD5. The FFA released by the above steps is transported to the mitochondria by FABP to activate UCP1 as well as to be burned as a substrate, eventually dissipating H+ potential energy through proton leakage and generating heat 48. Prolonged cold stimulation induces a large uptake of extracellular circulating lipids by brown adipocytes, which are mainly stored in the TG-rich lipoprotein (TRL) 50. However, brown adipocytes do not seem to be able to directly absorb, process and break down TRL particles. The main site of TRL breakdown is the endothelium adjacent to the adipocytes. The endothelial cells take up TRL via cluster of differentiation 36 (CD36) and then TRL is broken down by LAL 51. The released FAs are transported intracellularly via fatty acid transport protein 1 (FATP1) on the surface of brown adipocytes 52. Some of these FAs are transported to mitochondria for β-oxidation to promote thermogenesis through the synergistic action of carnitine palmitoyltransferase (CPT)1a and CPT2 transporter 53, while others are re-esterified to neutral lipids and stored in newly formed small LDs to alleviate lipotoxicity (**Figure 2**). The formation of small multilocular LDs in brown adipocytes increases the contact area between LD and lipase, which effectively promotes lipolysis and FAs transport to mitochondria close to LDs, thus accelerating mitochondrial β-oxidation and thermogenesis. Thus, thermogenesis is a precisely regulated process involving numerous factors. As the executioner of LD function, LD proteins play an important role in thermogenesis. The thermogenic effects of LD proteins can be divided into two categories: one regulating lipolysis and the other regulating the expression of UCP1, a key protein for thermogenesis.

In response to cold stimulation, sympathetic nerves are activated to release norepinephrine which binds to β-AR on brown and beige cells and dissociates the receptor-coupled trimeric Gs protein and activates AC, leading to cAMP synthesis. cAMP further activates PKA. PKA phosphorylates Plin1 leading to the release of CGI-58 sequestered by Plin1. CGI-58 interacts with FABP4 to facilitate ATGL activity to enhance lipolysis. PKA also phosphorylates HSL and causes its translocation to LD to further promote lipolysis. Cold stimulation induces FATP1 to transport FAs from the extracellular into brown adipocytes. A portion of FAs were transported to mitochondria for β-oxidation to promote thermogenesis through the synergistic action of CPT1α and CPT2 transport proteins, while another portion of FAs were re-esterified to neutral lipids for storage in newly formed small LDs to alleviate lipotoxicity.

### Lipid droplet proteins regulate lipolysis

Members of the PLIN family of intrinsic LD proteins, including Plin1-5, are important for regulating LD growth, maintaining LD dynamics 54 and thermogenesis. It has been shown that plin2 deficiency induces subcutaneous WAT browning in the groin at room temperature and activates the thermogenic program 55. Notably, a 20% sucrose diet induced subcutaneous WAT in plin2-deficient mice exhibiting significant browning and a significant increase in thermogenic gene expression, suggesting that Plin2 is a mediator of diet-induced adipose browning 55. Similarly, Plin3 deletion stimulates browning and thermogenic gene expression in inguinal white adipose tissue (iWAT) 56. Plin3-knockout mice exhibit enhanced cold tolerance and enhanced basal and stimulated lipolysis in iWAT, inducing activation of peroxisome proliferator-activated receptor alpha (PPARα). Plin3 deficiency promotes PPARα target gene and uncoupling protein 1 expression, and the formation of multi-compartment LD under cold stimulation 56. It is suggested that plin2 and plin3 serve as intrinsic protective factors that regulate adipocyte thermogenesis by limiting lipid metabolism and thermogenic gene expression. Plin5, a LD protein highly expressed in oxidative tissues 57, is involved in lipolysis regulation. Experimental data from mouse myoblasts suggest that Plin5 activates ATGL and promotes lipolysis by recruiting CGI-58 to the LD surface 58. However, another study showed that Plin5 interacts with ATGL lipolysis and inhibits LD breakdown 59. This suggests that Plin5 may have a two-sided role in the regulation of lipolysis. In addition, Plin5 is abundantly expressed in BAT and mediates LD-mitochondrial interactions, which are essential for BAT thermogenesis (we describe this in detail below).

Carboxylesterase 3 (Ces3) is a hydrolase with extensive activity in the liver and adipose tissue 60. Normally, ces3 is localized to the ER 61-63, however, it is transferred from the ER to LDs after cold exposure or isoproterenol (ISO) treatment and becomes the major LD-targeting protein 64. It has been shown that Ces3 translocation to LDs is dependent on PKA-activated pathways, but the precise mechanism by which PKA regulates Ces3 translocation between the two organelles remains to be further investigated. Under β-adrenergic or cold conditions, Ces3 acts as a non-classical lipid hydrolase for lipolysis and promotes BAT thermogenesis. Interfering with Ces3 expression or inhibiting Ces3 enzymatic activity decreased β-oxidation and mitochondrial biosynthesis-related genes, UCP1 expression, and oxygen consumption rate in differentiated 3T3-L1 cells. This suggests an important role of Ces3 in ISO or cold stimulation-induced enhancement of mitochondrial function and activation of the adipogenic thermogenic program 64. Mechanistically, Ces3 inhibition downregulates the expression of Ucp1 and peroxisome proliferator-activated receptor gamma coactivator 1-alpha (PGC-1α) via peroxisome proliferator-activated receptor γ (PPARγ), which attenuates the thermogenic program of adipocytes and leads to marked impairment in the ability of mice to protect their body temperature in the cold 64. However, how precisely Ces3 regulates the expression of Ucp1, e.g., the direct interaction factors downstream of Ces3, remains to be investigated in depth.

Vacuolar protein sorting 13C (VPS13C), a mammalian ortholog of yeast Vps13 65, 66, is highly expressed in mouse BAT 67. Biochemical experiments have shown that VPS13C is an LD-associated protein in adipocytes that is closely associated with the multilocular phenotype of adipocytes 67. Research data demonstrated that VPS13C has a suppressive effect on the expression of genes related to the regulation of LD catabolism and thermogenesis 67. Silencing of VPS13C decreased LD size and triglyceride content and increased the release of basal free FAs. Mechanistically, this is due to the deletion of vps13c, leading to an increase in ATGL, but not HSL-targeted LDs, which promotes LD hydrolysis. In addition, deletion of VPS13C upregulates thermogenesis-related genes, such as PPARα, PGC-1α, UCP1, and CIDEA, suggesting that VPS13C can inhibit thermogenesis 67. Furthermore, how VPS13C localizes to LDs remains unknown, and analysis of the VPS13C sequence revealed the presence of a pair of amphiphilic slices in the plane with the membrane, suggesting that VPS13C may have an intramembrane-anchoring structural domain and directly bind LDs. Previous studies have shown that yeast VPS13C homologs can mediate organelle interactions and that human VPS13C can interact with vesicle-associated membrane protein-associated proteins A and B (VAPA and VAPB) and is an ER protein known to mediate ER-organelle interactions and lipid transfer. Whether VPS13C regulates the interactions between LDs and other organelles in adipocytes and whether such interactions are associated with thermogenesis remains to be further investigated.

Hypoxia-inducible gene 2 (Hig2) is a protein containing 63 amino acids and is expressed in both adipose and liver tissues and is localized to the surface of LDs 68, 69. At 23°C, adipocyte-specific Hig2 deficiency altered the adipose tissue distribution in HFD-fed mice. Epididymal WAT (eWAT) body weight was significantly lower in Hig2AdKO animals fed a high-fat diet, indicating that Hig2-specific deficiency reduced fat storage deposition. However, at 30 °C, the phenomenon in which Hig2-specific deficiency reduced fat storage deposition disappeared. This indicates that Hig2-specific deficiency reduces fat storage and requires activation of non-shivering thermogenesis 69. But how hig2 deficiency promotes fat release requires further investigation.

### Lipid droplet proteins regulate the expression of thermogenic genes

**Figure 3:**
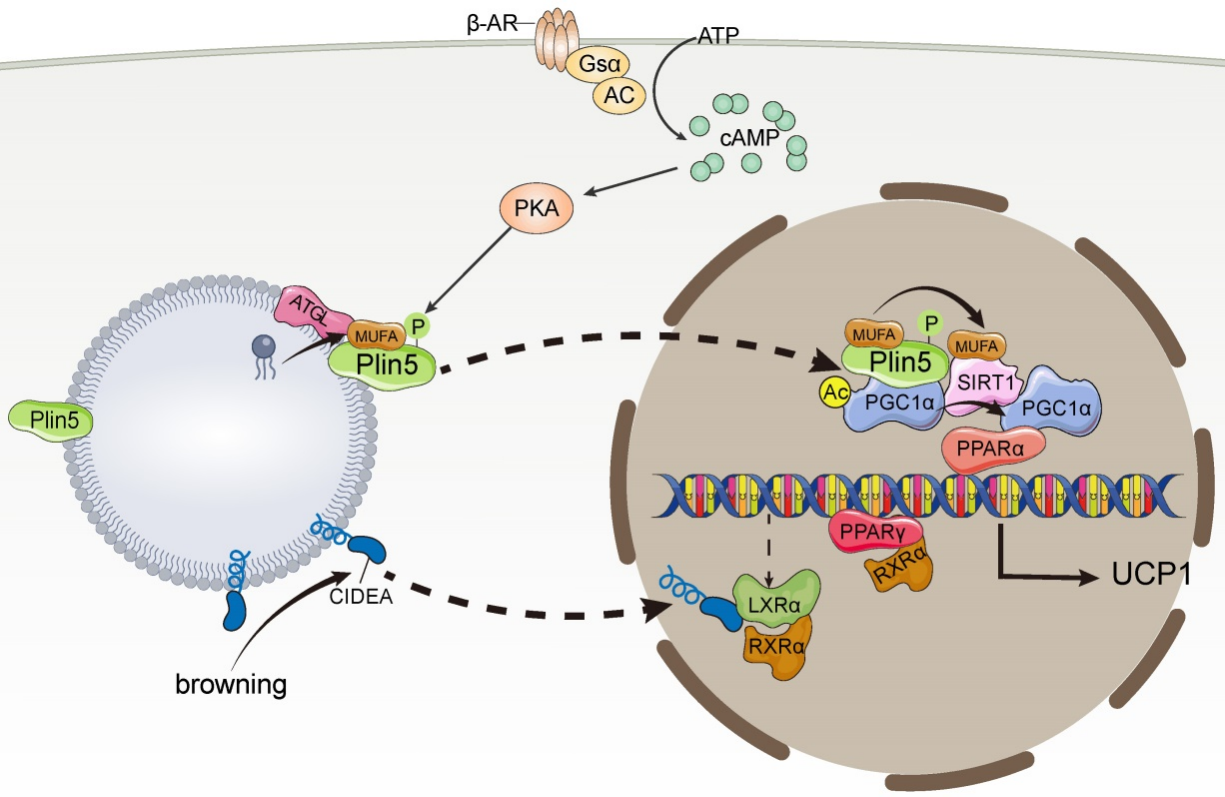
** Lipid droplet proteins regulate thermogenic gene expression.** MUFA, monounsaturated fatty acids; PPARα, peroxisome proliferator-activated receptor alpha; PPARγ, peroxisome proliferator-activated receptor γ; PGC-1α, peroxisome proliferator-activated receptor gamma coactivator 1-alpha; LXRα, Liver X Receptor alpha; SIRT, silencing information regulator factor 2-related enzyme 1.

CIDEA not only promotes LD fusion but also plays an important role in thermogenesis. CIDEA, which has a different expression profile in humans and mice, is only expressed in mouse BAT, whereas it is expressed in human WAT and BAT 69. In human adipocytes, CIDEA knockout downregulates the expression of thermogenic genes, such as PGC1α and UCP1, whereas re-expression of CIDEA reverses the downregulation of thermogenic genes caused by CIDEA deletion 69. In addition, CIDEA knockout reduced mitochondrial respiration, proton leakage, and maximal respiratory capacity of beige adipocytes. Mechanistically, during browning, CIDEA translocates from LDs to the nucleus and specifically binds to the Liver X Receptor alpha (LXRα), thereby attenuating the inhibition of UCP1 enhancer activity by LXRα and enhancing the binding of PPARγ to the UCP1 enhancer, which drives UCP1 transcription for thermogenesis 69. In addition, Transcriptome data analysis revealed genes participating in global thermogenic processes, for example, adaptive thermogenesis, fatty acid oxidation, mitochondrial transport, electron transport chain, were downregulated in CIP-deficient human beige adipocytes, indicating the control of thermogenic and metabolic functions by CIDEA 70 (**Figure 3**).

However, the function of CIDEA in the brown fat of mice differs considerably from that in humans. Under cold stimulation, brown fat in CIDEA-null mice exhibited a higher metabolic rate, body temperature, and lipolysis rate, suggesting greater thermogenic activity 71. Zhou et al. found that CIDEA localizes to mitochondria and cooperates with and suppresses the uncoupling activity of UCP1 71.

In another study using transgenic aP2-hCidea mice that overexpressed human CIDEA in all adipose tissues, UCP1 activity was significantly inhibited in BAT mitochondria isolated from aP2-hCidea mice 72. Interestingly, Fischer et al. found that CIDEA itself did not localize to the mitochondria, contradicting the findings of Zhou et al. 71. Fischer suggested that Zhou's findings may have resulted from contamination of membrane fractions during the experiment. Fischer suggested that CIDEA may indirectly inhibit UCP1 activity, while mice increased the total amount of UCP1 in the tissues to match the diminished thermogenic capacity of the UCP1 protein through a properly balanced compensatory mechanism 72.

Plin5 not only regulates lipolysis directly on the surface of LDs but also acts as a transcriptional coactivator in response to catecholamine stimulation and participates in lipolysis. Mechanistically, catecholamine facilitates PKA phosphorylation of Plin5, leading to the translocation of Plin5 from LDs to the nucleus and the formation of transcriptional complexes with PGC-1α and silencing information regulator factor 2-related enzyme 1 (SIRT1) 73, which deacetylates PPARγ in a ligand-dependent manner 74, 75. Plin5 binds to SIRT1 and disinhibits its deacetylase activity, which in turn promotes PGC-1α promoter activity to activate PGC-1α target genes, thereby promoting the transcription of thermogenesis-related mitochondrial biogenesis, and oxidative function-related genes, ultimately leading to lipolysis 73. Interestingly, catecholamine-activated PKA phosphorylation events promote Plin5 binding of LD-derived monounsaturated fatty acids (MUFAs) mediated by ATGL, which facilitates Plin5 nuclear translocation 76. Plin5-carried MUFAs induce conformational changes in SIRT1 in the nucleus by directly binding to SIRT1, thereby activating SIRT1 76. Ultimately, MUFAs enhance the transcriptional activity of PGC-1α in a SIRT1-dependent manner, which in turn promotes the expression of thermogenesis-related genes 76 (**Figure 3**).

Aifm2, a FAD-dependent NADH/NAD oxidoreductase, was identified as an LD-associated protein that is highly enriched in BAT 77. It was shown that Aifm2 in BAT and WAT promotes oxygen consumption, uncoupled respiration, and thermogenesis during cold and diet-induced thermogenesis and plays a key role in glycolysis and glucose oxidation 77. In response to cold stimulation or β-adrenergic stimulation, Aifm2 translocates from LDs to the outer mitochondrial inner membrane, maintains high cytoplasmic NAD levels by oxidizing NADH, and maintains a stable rate of glycolysis and glucose oxidation 77. Aifm2 increases overall mitochondrial activity in BAT cells and transfers electrons to the electron transport chain, providing fuel for thermogenesis and promoting uncoupled respiration and thermogenesis 77. However, there are many questions regarding the role of Aifm2 in thermogenesis that require further investigation, e.g., the molecular mechanism of Aifm2 transfer from the LDs to mitochondria in response to cold stimulation.

Cold exposure stimulates ATGL-mediated lipolysis and production of MUFA. Plin5 phosphorylated by PKA preferentially binds MUFA and translocates to the nucleus. MUFA enhances PGC-1α/PPARα signaling by activating SIRT1 and promotes mitochondrial oxidative metabolism and UCP1 expression. During the induction of browning, CIDEA is transferred from lipid droplets to the nucleus in a concentration-dependent manner. CIDEA binds LXRα and inhibits its repression of UCP1 enhancer RXRα activity, thereby enhancing PPARγ activity and activating UCP1 transcription.

## Lipid droplet-mitochondrion contacts to regulate thermogenesis

**Figure 4:**
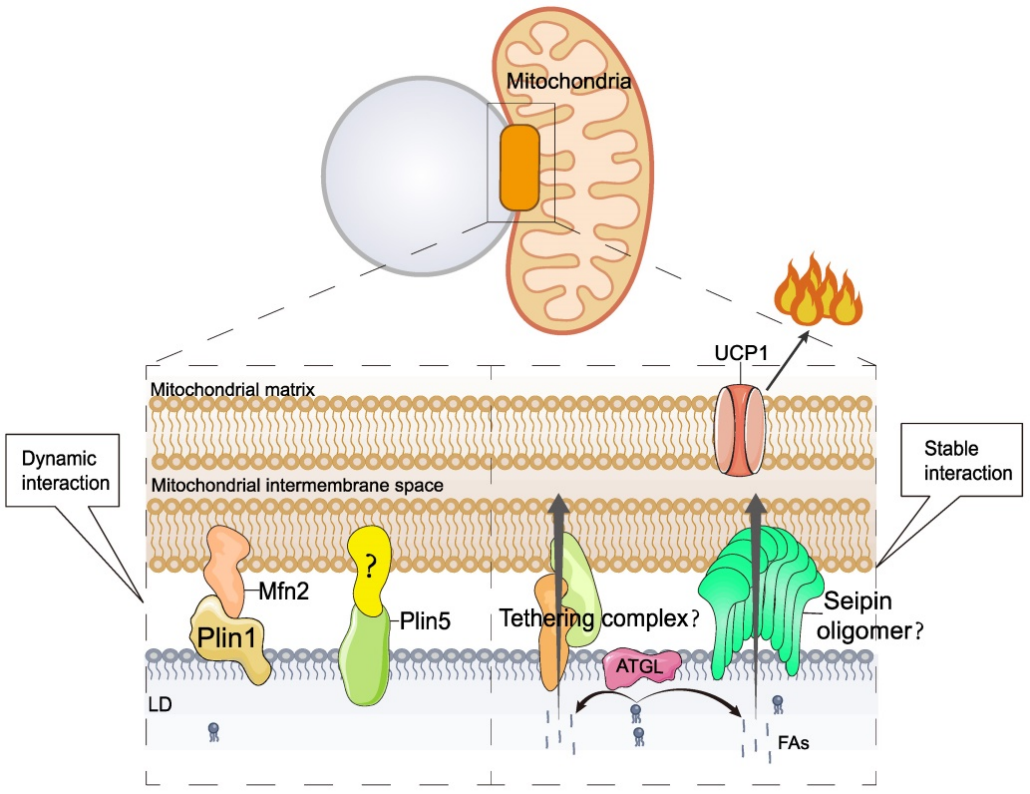
** Mechanism of lipid droplet-mitochondrial interactions in thermogenesis.** Mfn2, mitofusin 2.

In addition to the presence of multilocular LDs, the most characteristic feature of thermogenic adipocytes is the high content of mitochondria and the consequent brown and beige coloration. Numerous studies have shown that there is an extensive network of interactions between LDs and mitochondria 78, 79 in a variety of oxidative tissues, including brown adipose tissue 80, 81, the heart 82, and type I skeletal muscle 83. LD-mitochondrial interactions can be subdivided into two categories according to their strength: (I) dynamic interactions, which can be disrupted by high-speed centrifugal forces (9,000×g). Benador et al. 78 used 9,000×g centrifugal force to isolate mitochondria from mouse brown adipocytes that interact with LDs and named them peridroplet mitochondria (PDM). (II) Stable interactions that could not be disrupted by high-speed centrifugation (228,000×g). Cui et al. 81 used ultracentrifugation at 228,000×g, which also failed to separate LDs from mitochondria; these were named LD-anchored mitochondria (LDAM) (**Figure 4**). Organelle complexes formed by anchoring LDs to the mitochondria are considered to be permanently intercalated.

Under cold stimulation, LDs-mitochondrial contact is increased in BAT, which is thought to facilitate the rapid consumption of fat and heat production 80, 84. The interaction between LDs and mitochondria is precisely regulated, and studies have shown that several proteins are involved in regulating LD-mitochondrion interactions, including mitofusin 2 (Mfn2) 85, Plin5, and others. In BAT, Mfn2 regulates LD-mitochondrial interactions by specifically interacting with Plin1, and this interaction requires Mfn2 GTPase activity 85. Mfn2-regulated LD-mitochondrial interactions respond to cold or adrenergic stimulation. Adipose-specific Mfn2 knockout mice (Mfn2-adKO) of BAT exhibit higher lipid accumulation and larger LDs 85. Lipid accumulation was not due to an increase in lipid synthesis genes but to a decrease in the rate of lipolysis. In addition, the BAT mitochondria of Mfn2-adKO animals showed significantly reduced levels of complexes I and III, which inhibited the respiratory capacity of BAT. Impaired lipolysis and oxidative capacity result in reduced thermogenesis in Mfn2-adKO mice, which are unable to maintain body temperature during cold exposure 85.

Plin5, a LD protein, regulates LD-mitochondrial interactions. Cold stimulation induces elevated BAT Plin5 expression, which promotes BAT FA uptake, mitochondrial biogenesis, cristae packaging, and oxidative function, thereby enhancing UCP1-dependent mitochondrial respiration and thermogenesis 86. Plin5 overexpression in BAT also leads to increased insulin sensitivity and reduced inflammation in iWAT 86. In addition, Plin5 knockdown reduces mitochondrial oxygen consumption rate and mitochondrial DNA content 69.

Interactions between LDs and mitochondria are widely believed to facilitate the rapid entry of FAs into the mitochondria. However, the mechanism by which FAs enter mitochondria from LDs is poorly understood. Seipin can form nanoscale tubular interfaces that facilitate the flow of lipids from the ER into nascent LDs 20, 21, 23, 24. Interestingly, recent studies have found that seipin is localized at the contact site between LDs and mitochondria 22. Therefore, seipin may regulate LD-mitochondrial interactions and mediate the transfer of FAs from LDs to mitochondria. However, induction of cAMP/PKA signaling in the absence of Bscl2 results in increased lipolysis and fatty acid oxidation in WAT and BAT, as well as browning of WAT 87, suggesting a potential inhibition of lipolysis and thermogenesis by seipin, which does not seem to support the above hypothesis. In conclusion, the mechanism of FA transfer between LDs and mitochondria requires further investigation.

Cold stimulation induces significant LD-mitochondrial interactions in thermogenic adipose tissue. Two mechanisms of LD-mitochondrial interactions have been identified. I) Dynamic Interaction. This process is regulated by the Plin1-Mfn2 complex and Plin5; however, the presence of the Plin5 interaction factor is unclear. II) Stable Interaction. This process is known as permanent LD-mitochondrial interaction or LD-mitochondrial anchoring. The molecular mechanism of LD-mitochondrial anchoring is poorly understood. Seipin, a protein with a lipid transport channel structure, is found to localize to the LD-mitochondrial contact site. Therefore, it is hypothesized that seipin may mediate LD-mitochondria interactions and direct FA transport between LDs and mitochondria.

## Lipophagy regulates adipose thermogenesis

Autophagy, an important pathway for degrading cellular contents to maintain normal cellular function and dynamic homeostasis 88, has also been found to be involved in LD degradation 89-91. The degradation of LDs by autophagy is mainly dependent on acidic hydrolases of lysosomes such as the LIPA/LAL (lipase A, lysosomal acid), and the process of autophagy-mediated LD degradation is called lipophagy which is divided into three main pathways: macrolipophagy, microlipophagy, and chaperone-mediated autophagy 89-93.

Macrolipophagy begins with the segregation of LDs by autophagosomes, which are then delivered to lysosomes for turnover 94. Macrolipophagy consists of microtubule-associated protein 1A / 1B-light chain 3 (LC3-II)-dependent phagocytosis of some LDs to form lipoautophagosomes, which subsequently fuse with lysosomes to form autolysosomes 94. During microlipophagy, LDs and lysosomes undergo lipid transfer via transient contact 95, 96. Chaperone-mediated autophagy facilitates the transfer of LD proteins Plin2 and Plin3 from HSP70 to lysosomes for degradation, thereby increasing the accessibility of cytoplasmic lipases to LDs 97, 98.

**Figure 5:**
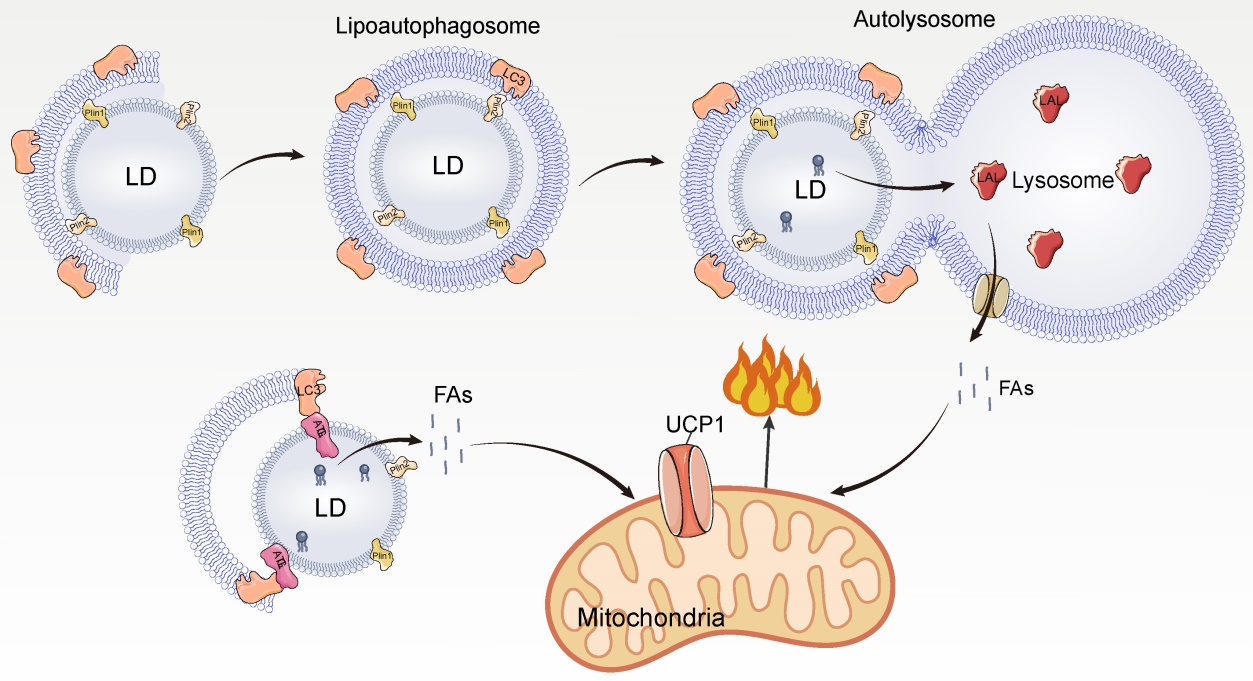
** Mechanism of lipophagy mediated thermogenesis.** LAL, lysosomal acid lipase.

In BAT, cold stimulation activates autophagy in hypothalamic pro-opiomelanocortin (POMC) neurons and lipophagy in BAT via neural activation, promoting LC3 localization to LDs and formation of lipoautophagosomes, leading to lipophagy and promoting FA utilization 99 (**Figure 5**). Inhibition of autophagy of POMC or BAT leads to impaired BAT lipophagy, reduced uncoupled respiration rate, and lipid accumulation in mice, making them unable to maintain normal body temperature under cold exposure 99. This indicates that BAT lipophagy has an important role for thermogenic function in mice. In addition, the interface between LDs and autophagosomes provides a platform for LC3 to interact with lipolytic enzymes, and LC3 interacts with ATGL to promote lipolysis 99. Under cold stimulation, lipophagy and lipolysis have complementary effects on FA mobilization.

Whether microautophagy occurs in BAT and promotes lipolytic and thermogenic functions remains unclear. Notably, both macroautophagy and microautophagy are extremely important for the regulation of lipid metabolism in the liver 94, 96, suggesting that macroautophagy and microautophagy can function in the same tissue. Microautophagy-mediated transient interactions between lysosomes and LDs rapidly degrade neutral lipids, which is important for the rapid mobilization of lipolysis. In response to cold stimulation, the body can rapidly respond and mobilize lipids; therefore, microautophagy may occur in BAT to rapidly mobilize lipids.

Smaller LDs have higher surface resolution and are more conducive to lipolysis. In the liver, larger LDs are reduced by ATGL lipolysis, and smaller LDs are eliminated by lipophagy 90. In BAT, LDs may need to be smaller for rapid lipolysis. Current studies suggest that there are two main ways in which LDs become smaller: one is a lipolytic process involving lipolytic enzymes 93, 100 and the other is the generation of smaller LDs by budding from larger LDs 101. The process of LD budding has been demonstrated *in vitro*, but it remains to be confirmed whether it occurs abundantly and rapidly *in vivo* in response to lipolysis. WAT has unilocular LDs that become multilocular after browning, and how unilocular LDs are converted to multilocular LDs is not known. LD budding provides the possibility for browning. Thus, LD budding may play an important role in rapid lipid utilization and browning of WAT.

Cold stimulation strongly activates lipophagy in BAT. The currently identified lipophagy in BAT is macroautophagy. Microtubule-associated protein 1A/1B-light chain 3 (LC3-II)-mediated macroautophagy engulfs LD to form a lipoautophagosome, which later fuses with lysosomes leading to TAG hydrolysis by lysosomal acid lipase (LAL) to hydrolyze and release FAs. In addition, LC3-II can directly interact with ATGL on the surface of LDs and promote lipolysis.

## The role of lipid droplets in the futile cycle of thermogenesis

Futile metabolic cycling mechanisms, in which one ATP-consuming reaction takes place concurrently with an inverse energetic reaction, are the basis of UCP1-independent thermogenesis. Other than ATP depletion and energy loss in the form of heat, these futile cycles have no overall effect. Previous studies have shown the existence of three futile cycling mechanisms, one being the Ca2+ cycle thermogenesis 10. The second is the creatine cycling, which is related to phosphorylation and dephosphorylation of creatine 102. There is no direct relationship between these two futile cycling mechanisms and LDs. The third one is the futile cycle between TAG and FAs 103, 104. TAG is catalyzed by lipase and eventually forms glycerol and FAs, which in turn can be re-esterified to TAG. This futile cycle can be induced by thiazolidinediones (TZDs) 103, cold exposure 104, and leptin 105 by promoting lipolysis. Therefore, LD proteins involved in lipolysis such as ATGL, Plin1, and CGI-58, Plin5 may have an effect on this cycle. The LD-mitochondrial interface is considered to be a good site for metabolic reactions, and LD-mitochondrial interactions are not only involved in lipolysis but also promote LD expansion by facilitating triglyceride synthesis 78. Therefore, LD-mitochondrial interactions may be involved in the ineffective cycle of TAG, but experimental data are lacking to support this hypothesis.

## Concluding Remarks

Although the role of LDs in thermogenesis has been well studied, there are still many unanswered questions. For example, it is still unknown which protein mediates the anchoring of LDs to mitochondria. It has been shown that Plin5 mediates LD-mitochondrial interactions, but deletion of Plin5 does not affect LD-mitochondrial anchoring. In addition, the mechanism of FA transfer between LDs and the mitochondria remains unknown. Direct FA transfer between LDs and the mitochondria is thought to be beneficial in alleviating cellular lipotoxicity. This direct transfer of FAs in organelles may be directly regulated by proteins but may also be a direct result of membrane fusion. In conclusion, it would be of great interest to explore the mechanism of FA transfer between LD and mitochondria.

Previous studies have shown that LDs of different sizes in the liver have different protein compositions, indicating different functions and fates. Large LDs in the liver are subjected to ATGL-mediated lipolysis and become smaller, while small LDs are degraded via the autophagic pathway. Brown or beige fat contains multilocular LDs, and there are differences in size between LDs. The protein composition on the surface of LDs of different sizes isolated from BAT differs; however, it remains unknown whether differences between LD proteins affect lipolysis as well as the function of LDs. In summary, a detailed understanding of LD-organelle interactions and LD dynamics will help in understanding the role of LDs in thermogenesis.

## Abbreviations

UCP1: uncoupling protein 1
LDs: lipid droplets
WAT: white adipose tissue
BAT: brown adipose tissue
TAG: triglycerides
FAs: fatty acids
LDAF1: lipid droplet assembly factor 1
FIT2: fat storage-inducing transmembrane protein 2
GPAT4: lycerol-3 phosphate acyltransferase 4
DGAT2: acyl-CoA:diacylglycerol acyltransferase
CIDEA: cell death-inducing DFF45-like effector family proteins A
CIDEB: cell death-inducing DFF45-like effector family proteins B
CIDEC: cell death-inducing DFF45-like effector family proteins C
LDCS: lipid droplets contact sites
PA: phosphatidic acid
FFA: free fatty acids
HDAC6: histone deacetylase 6
Plin1: perilipin 1
DAG: diacylglycerol
PAP: phosphatidic acid phosphatase
GTP: Guanosine triphosphate
GDP: guanosine diphosphate
PUFA: polyunsaturated fatty acids
LLPS: liquid-liquid phase separation
ATGL: adipose triglyceride lipase
HSL: hormone-sensitive lipase
NA: norepinephrine
BAT: brown adipose tissue
β-ARs: β-adrenoceptors
PKA: protein kinase A
AC: adenylate cyclase
iWAT: white adipose tissue
PPARα: peroxisome proliferator-activated receptor alpha
Ces3: Carboxylesterase 3
ISO: isoproterenol
PPARγ: peroxisome proliferator-activated receptor γ
PGC-1α: peroxisome proliferator-activated receptor gamma coactivator 1-alpha
Hig2: Hypoxia-inducible gene 2
LXRα: Liver X Receptor alpha
SIRT1: silencing information regulator factor 2-related enzyme 1
MUFAs: monounsaturated fatty acids
PDM: peridroplet mitochondria
LDAM: LD-anchored mitochondria
Mfn2: mitofusin 2
POMC: pro-opiomelanocortin
LAL: lysosomal acid lipase
FATP1: fatty acid transport protein 1
CPT1a: carnitine palmitoyltransferase 1A
CPT2: carnitine palmitoyltransferase 2
